# Evolution of bilaterian central nervous systems: a single origin?

**DOI:** 10.1186/2041-9139-4-27

**Published:** 2013-10-07

**Authors:** Linda Z Holland, João E Carvalho, Hector Escriva, Vincent Laudet, Michael Schubert, Sebastian M Shimeld, Jr-Kai Yu

**Affiliations:** 1 Marine Biology Research Division, Scripps Institution of Oceanography, University of California at San Diego, La Jolla, CA 92093-0202, USA; 2 Laboratoire de Biologie du Développement de Villefranche-sur-Mer (UMR 7009 – CNRS/UPMC), Observatoire Océanologique de Villefranche-sur-Mer, 181 Chemin du Lazaret, B.P. 28, 06230 Villefranche-sur-Mer, France; 3 CNRS, UMR 7232, BIOM, Université Pierre et Marie Curie Paris 06, Observatoire Océanologique, 66650 Banyuls-sur-Mer, France; 4 Institut de Génomique Fonctionnelle de Lyon (CNRS UMR5242, UCBL, ENS, INRA 1288), Ecole Normale Supérieure de Lyon, 46 allée d’Italie, 69364 Lyon Cedex 07, France; 5 Department of Zoology, University of Oxford, The Tinbergen Building, South Parks Road, Oxford OX1 3PS, UK; 6 Institute of Cellular and Organismic Biology, Academia Sinica, Taipei 11529, Taiwan

**Keywords:** Central nervous system evolution, Hemichordate, Urbilaterian, Amphioxus, Tunicate, Vertebrate brain, Nerve cord

## Abstract

The question of whether the ancestral bilaterian had a central nervous system (CNS) or a diffuse ectodermal nervous system has been hotly debated. Considerable evidence supports the theory that a CNS evolved just once. However, an alternative view proposes that the chordate CNS evolved from the ectodermal nerve net of a hemichordate-like ancestral deuterostome, implying independent evolution of the CNS in chordates and protostomes. To specify morphological divisions along the anterior/posterior axis, this ancestor used gene networks homologous to those patterning three organizing centers in the vertebrate brain: the anterior neural ridge, the zona limitans intrathalamica and the isthmic organizer, and subsequent evolution of the vertebrate brain involved elaboration of these ancestral signaling centers; however, all or part of these signaling centers were lost from the CNS of invertebrate chordates. The present review analyzes the evidence for and against these theories. The bulk of the evidence indicates that a CNS evolved just once – in the ancestral bilaterian. Importantly, in both protostomes and deuterostomes, the CNS represents a portion of a generally neurogenic ectoderm that is internalized and receives and integrates inputs from sensory cells in the remainder of the ectoderm. The expression patterns of genes involved in medio/lateral (dorso/ventral) patterning of the CNS are similar in protostomes and chordates; however, these genes are not similarly expressed in the ectoderm outside the CNS. Thus, their expression is a better criterion for CNS homologs than the expression of anterior/posterior patterning genes, many of which (for example, *Hox* genes) are similarly expressed both in the CNS and in the remainder of the ectoderm in many bilaterians. The evidence leaves hemichordates in an ambiguous position – either CNS centralization was lost to some extent at the base of the hemichordates, or even earlier, at the base of the hemichordates + echinoderms, or one of the two hemichordate nerve cords is homologous to the CNS of protostomes and chordates. In any event, the presence of part of the genetic machinery for the anterior neural ridge, the zona limitans intrathalamica and the isthmic organizer in invertebrate chordates together with similar morphology indicates that these organizers were present, at least in part, at the base of the chordates and were probably elaborated upon in the vertebrate lineage.

## Review

### Introduction

There is general agreement that the relatively complex central nervous system (CNS) characterizing most higher metazoan animals can be traced back through evolution to a nerve net in a cnidarian-like ancestor. However, it is highly controversial whether the nervous system of the next evolutionary stage (the urbilaterian) still consisted solely of a nerve net or included a CNS. If the urbilaterian had only a nerve net, then the CNSs of protostomes and deuterostomes likely evolved independently. In contrast, the view that the urbilaterian had a CNS is consistent with the view that the CNSs of all metazoans are homologous. At present, opinion is still divided, with the majority advocating a single evolutionary origin for the CNS [1-8] and the minority favoring an urbilaterian with a nerve net [9,10].

This controversy about CNS evolution is intimately related to issues of homology, and it is useful to outline current thinking about homology at the outset. It is important not to conflate the concepts and recognition criteria for homology. There are currently three concepts underlying homology – biological [11], taxic/cladistic [12,13] and historical [14-16]. What matters for the last, which is the most familiar and most germane for the nervous system controversy, is the historical continuity of descent from a common ancestor. The historical concept requires one to be explicit about what is being compared [17]; for example, bird wings and bat wings are homologous as vertebrate forelimbs, but not as wings. Importantly, historical homologies can become very different through divergence. The three chief criteria for recognizing homology are relative position to other body parts, special quality, and transitional stages [18]. A developmental criterion, introduced by Haeckel [19], proved difficult to apply and has been largely submerged into the previous three criteria. Importantly, there are differing views concerning the hierarchical distribution of homology across levels of biological organization. In the view of Striedter and Northcutt, homology at one level (say behavior) does not necessarily connote homology at another (say morphology) [20]. In contrast, Wagner argued that structures descended from a common ancestor are homologous even if they have diverged and have no clear morphological similarity [16]. The problems raised by the hierarchical nature of homology have been heightened by the discovery of developmental gene conservation [21] and are especially noticeable in the discussion of CNS evolution.

**Figure 1:**
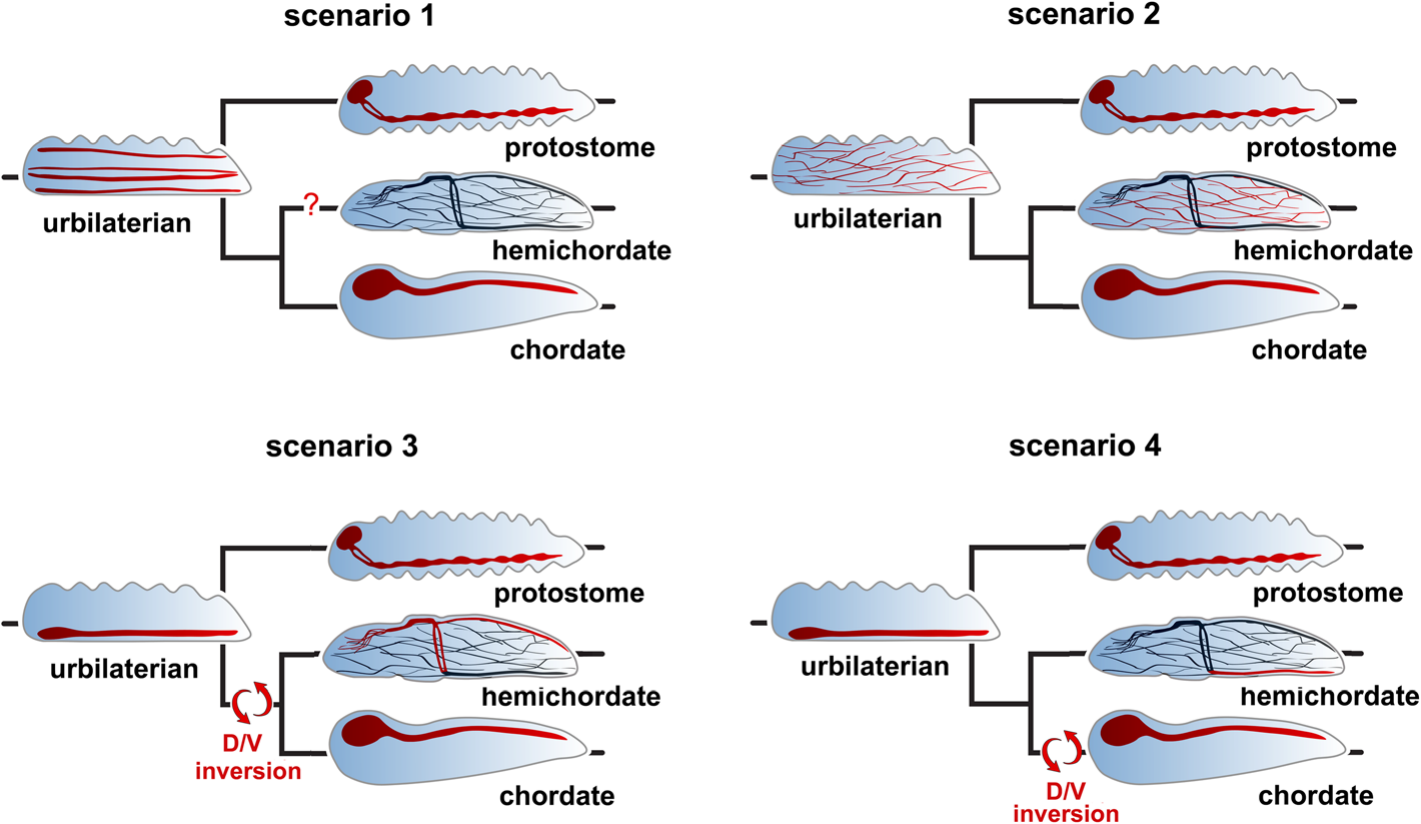
**Four scenarios for evolution of central nervous systems in bilaterians.** In scenario 1, the urbilaterian had multiple nerve cords, one of which evolved into the dorsal central nervous system (CNS) of chordates, while another nerve cord evolved into the ventral CNS of protostomes. In scenario 2, the CNSs of protostomes and deuterostomes evolved independently from an ectodermal nerve net in the bilaterian ancestor. In scenario 3, the chordate and protostome nerve cords evolved from a ventral nerve cord in the urbilaterian ancestor. A dorso/ventral (D/V) inversion occurred at the base of the deuterostomes; the dorsal nerve cord of hemichordates is thus homologous to the chordate CNS and to the protostome ventral nerve cord. In scenario 4, the protostome and chordate nerve cords evolved from the CNS of an urbilaterian ancestor, but a D/V inversion occurred at the base of the chordates. Thus, the ventral nerve cord of a hemichordate is homologous to the chordate and protostome CNSs. Scenarios after [1,3,7,23,26-29].

In the past 20 years, it has been found that developmental genes and core signaling pathways are typically conserved across phyla and that gene expression patterns during development can often be used as characters for inferring homologies. Thus, although the majority view had been that the urbilaterian had a nerve net, the balance was tipped towards an urbilaterian with a CNS by the discovery that *bone morphogenic protein* (*BMP*)*/decapentaplegic* genes were expressed dorsally in *Drosophila* and ventrally in vertebrates with the BMP antagonists *chordin/short gastrulation* expressed on the opposite side [22] (Figure 1). In this view, which is consistent with the CNSs of all higher metazoans being homologous, a dorso/ventral (D/V) inversion occurred either in basal protostomes or in the deuterostome lineage [3,5,23]. However, in the last 10 years, studies of gene expression and function in an enteropneust (acorn worm; phylum Hemichordata) have been interpreted as evidence that the ancestral deuterostome and, by extension, the urbilaterian had a nerve net and no CNS [9,24,25]. Thus, while a CNS would have arisen close to the base of the protostomes, the evolution of a CNS in deuterostomes did not occur until the base of the chordates. In the present review, we examine the detailed evidence on both sides of the controversy and evaluate its interpretations. We conclude that a stronger case can be made for the initial appearance of the CNS at the level of the urbilaterian than for independent evolution of the CNS in more than one line of metazoan descent.

### Reconstructing the ancestral bilaterian

Although several features of the ancestral bilaterian in addition to the presence or absence of a CNS are widely debated, a range of molecular, developmental and comparative morphological evidence indicates that this animal was bilaterally symmetrical, with distinct anterior and posterior ends, dorsal and ventral surfaces, and left and right sides. It almost certainly had defined muscle, derived from mesoderm, allowing active locomotion and a gut with either a single opening or a separate mouth and anus [30]. Whether or not this animal had a CNS, an ectodermal nerve net or some combination of the two has been hotly debated (reviewed in [31]) (Figure 1).

One difficulty in deciding whether the ancestral bilaterian had a CNS is that the ectoderm in bilaterians is broadly neurogenic. Therefore, the distinction between the CNS and the remainder of the relatively neurogenic ectoderm is not always clear-cut. In chordates, arthropods and annelids, the distinction is most clear as there is a fully internalized concentration of neurons, axons and supporting cells along the anterior/posterior (A/P) axis (that is, a CNS) that integrates information from sensory cells both associated with the CNS (for example, eyes) and with other portions of the ectoderm and coordinates behavior. Importantly, the CNS in these organisms has an anterior concentration of discrete neural centers or “brain”, which coordinates sensory inputs and responses. At the other extreme are “diffuse ectodermal nerve nets” such as in cnidarians. However, such nerve nets are not uniform; specific types of neurons may be regionally localized [32]. An additional problem in understanding the evolution of CNSs comes with the Ambulacraria (echinoderms and hemichordates), as they have both ectodermal nerve nets and nerve cords. It is controversial whether echinoderm and/or hemichordate nerve cords, neither of which has a concentration of neurons that could be termed a brain, and the CNS of chordates have a common evolutionary origin [33,34]. Here we will use the term CNS for a nervous system that is derived from ectoderm, includes both axons and neurons and is specialized along the A/P axis with an anterior concentration of neural centers (brain), and the term “nerve cord” more broadly to include axonal tracts with few or no neurons and lacking a discrete brain. The diversity of animal nervous systems and paucity of data from some species may blur this distinction on occasion; however, we will be explicit in such instances.

#### What is the evidence for a CNS in the ancestral bilaterian?

**Figure 2:**
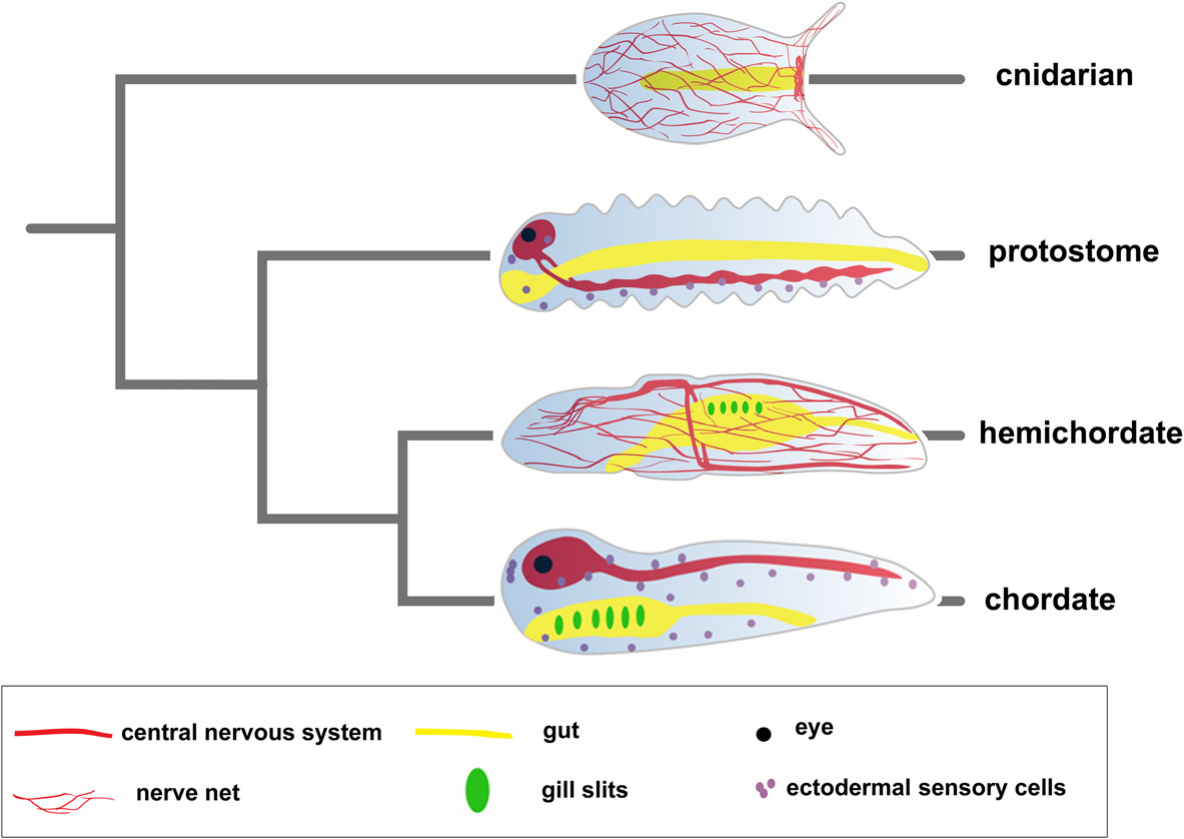
**Comparison of metazoan body plans.** A typical cnidarian polyp, a generalized protostome, hemichordate and chordate and their phylogenetic relations are shown. Special attention is given to nervous systems and neural structures of the respective animals.

It is generally agreed that bilaterians evolved from radially or bi-radially symmetrical animals, comparable in some ways to modern cnidarians. Adult cnidarians have an ectodermal nerve net with a concentration of neurons around the single gut opening (Figure 2). Therefore, if the ancestral bilaterian had already evolved a CNS, it would presumably have arisen as a concentration or amplification of neurons along one side of this nerve net, perhaps together with a reduction in numbers of neurons elsewhere in the ectoderm.

Unfortunately, no extant animal is a good stand-in for this ancestral bilaterian. Extant animals that are thought to have diverged from the bilaterian lineage before it radiated into the protostomes (Ecdysozoa and Lophotrochozoa) and the deuterostomes do not show an intermediate condition between a nerve net and a CNS (Figure 2). The best candidates for such early bilaterian offshoots are the acoel and nemertodermatid flatworms, and the xenoturbellids, which in some studies have been placed basal to the deuterostomes plus protostomes but in others are placed basal in the deuterostome lineage [35,36]. Acoels have a concentration of neurons, or a “brain”, anteriorly with up to six tracts of axons extending posteriorly [37]. In contrast, xenoturbellids have an intraepithelial nerve net that lacks aggregations of neurons or axonal tracts [38]. As a result of the lack of a clear intermediate, scenarios for evolution of CNSs are necessarily based on similarities in gene expression and neuroanatomy in the two main lineages of bilaterians: protostomes (Ecdysozoa plus Lophotrochozoa) and deuterostomes (Figure 1).

#### Regionalization of nerve cords in protostomes and chordates

Because the CNSs in protostomes and deuterostomes are in different positions, develop rather differently and are morphologically somewhat diverse, possible homologies between them have been highly contentious. Complicating the picture is that some of the genetic mechanisms for specifying A/P positions in the CNS are common to the entire organism, including the general ectoderm exterior to the CNS, and are therefore not entirely useful for inferring homologies of CNSs. For example, some genetic mechanisms mediating A/P patterning in the CNS were clearly inherited from a cnidarian-like ancestor in which they patterned the entire body axis. Thus, *Six3/6* and *Irx* are expressed in the aboral region of the planula larva of the sea anemone *Nematostella vectensis*, opposite the blastopore [39] and in the anterior end of the brain of both protostomes and deuterostomes – *Six3/6* in the anterior tip of the CNS and *Irx* genes a little more posteriorly [24,40-44]. In *N. vectensis* the domains of these two genes are initially congruent, while in the CNS of bilaterians the *Six3/6* domain is anterior to that of *Irx*. Therefore, although it is most parsimonious to propose that these genes were coopted into the CNS of an ancestral bilaterian, it cannot be ruled out that they were coopted independently into the CNS of protostomes and deuterostomes.

*Hox* genes are another example of A/P patterning genes that are not entirely useful for inferring homologies between the protostome and chordate CNS. The problem is that although they do mediate A/P patterning of the CNS in bilaterians [45,46], they mediate A/P patterning of other tissues as well [47-54]. Thus, while their expression patterns have been used to infer homologies between the CNS in insects and vertebrates, it remains possible that they patterned the entire body axis of the Urbilaterian and were independently coopted into the CNSs of protostomes and deuterostomes. It is not clear when a role for nested expression of *Hox* genes in regionalization of the A/P axis evolved. They do not appear to be involved in A/P patterning in cnidarians [55,56]. Comparisons of *Hox* genes in protostomes with up to 10 or 11 *Hox* genes and invertebrate deuterostomes with up to 15 indicate that the ancestral bilaterian had at least eight to 10 *Hox* genes [57], while cnidarians have up to six depending on the species and acoel flatworms have three, which are more or less regionally expressed in the surface ectoderm along the A/P axis [58,59] with later expression in putative neural precursors [49]. Thus, acoels either arose before a large *Hox* cluster evolved or they lost some *Hox* genes. Even so, a role for an expanded array of *Hox* genes in specification of A/P positions in the ectoderm was evidently present in the ancestral bilaterian. Thus, although expression of the *Drosophila melanogaster Hox1* gene *labial* in a stripe at the posterior end of the tritocerebrum within the *unpg* (*Gbx*) domain has been likened to nested expression of *Hox* genes in the vertebrate hindbrain, with *Hoxb1* being expressed in a stripe in rhombomere 4, the possibility that *Hox* genes were independently coopted into the CNS in protostomes and deuterostomes cannot be ruled out.

Stronger support for a single origin of the CNS comes from similar expression in the CNSs of protostomes and chordates of genes that are not expressed in comparable patterns in other tissues. Thus, Reichert and colleagues used gene expression patterns to support the perhaps surprising idea that the three parts of the *Drosophila* brain – protocerebrum, deutocerebrum and tritocerebrum [5,45] – are homologous to the forebrain, midbrain and hindbrain of vertebrates (Figure 2) [60]. For example, in *D. melanogaster*, the *Otx* homolog *Otd* is expressed throughout the protocerebrum and deutocerebrum, while *unpg* (homologous to *Gbx*) is expressed in the tritocerebrum, the subesophageal ganglion and the ventral nerve cord [5]. The domains of the two abut at the boundary between the deutocerebrum and tritocerebrum, similar to the abutting domains of *Otx2* and *Gbx2* at the midbrain/hindbrain boundary (MHB) in vertebrates [5,61]. In addition, although some domains of *Pax2/5/8* genes are not similar between the CNS of flies and chordates, *Pax2/5/8* is expressed at high levels in the posterior part of the deutocerebrum (just anterior to the deutocerebrum/tritocerebrum boundary) in *D. melanogaster*, while the three vertebrate *Pax2/5/8* genes are expressed at high levels at the MHB [5,62]. Moreover, in third instar larvae of *D. melanogaster*, the *earmuff* gene (homologous to *Fezf*) is broadly expressed in the anterior brain with a posterior boundary at the protocerebrum/deutocerebrum boundary [63]. The domain is just anterior to that of *mirror*, one of the three *Irx* homologs. Similarly, Irimia and colleagues showed that in chordates, the posterior limits of *Fez* genes (*Fez* and *Fez-like*) abut the anterior limit of *Irx1* in the forebrain [64]. In vertebrates, this is the zona limitans intrathalamica (ZLI) [65].

**Figure 3:**
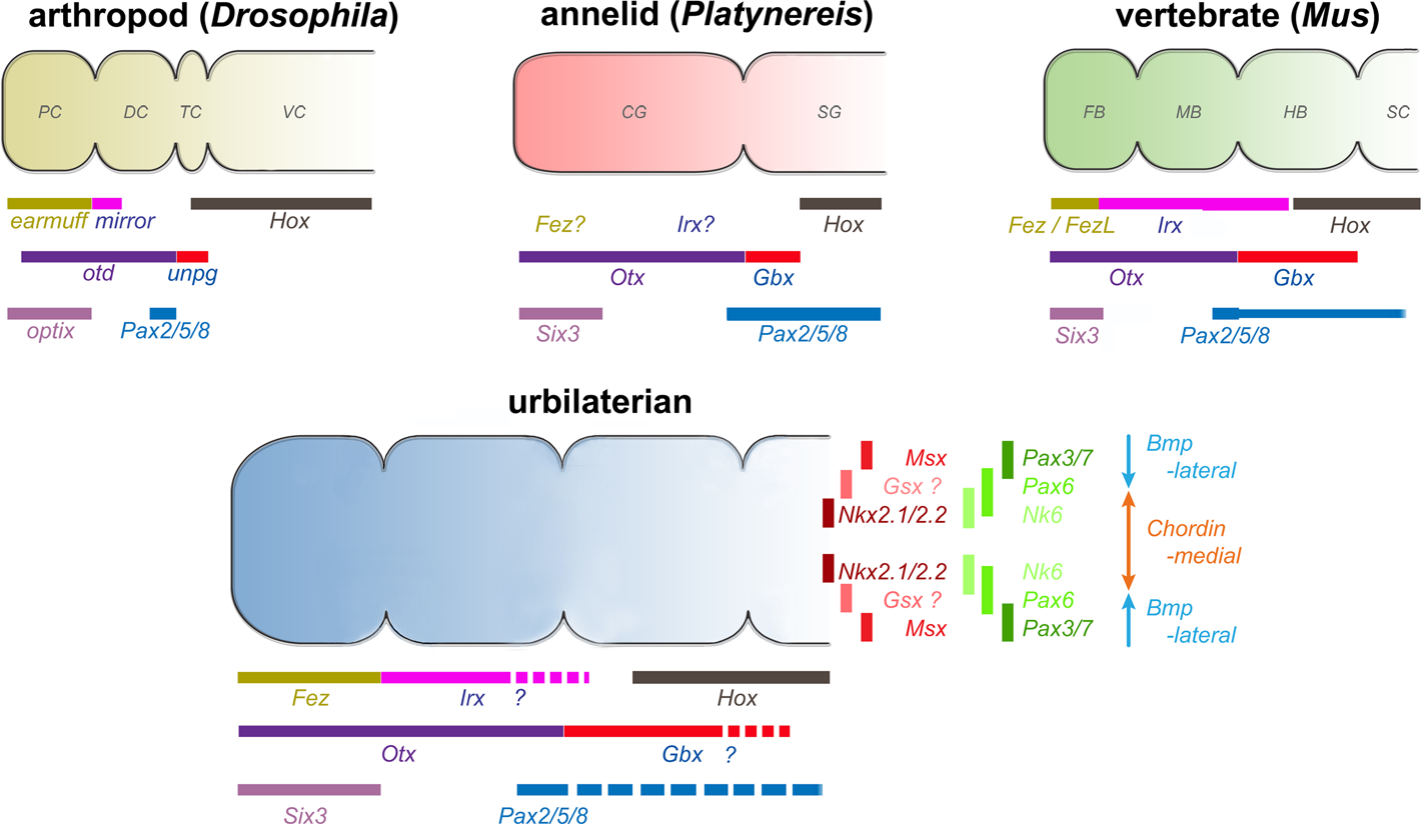
**Anterior–posterior gene expression in central nervous systems of three extant bilaterians and the urbilaterian.** Anterior–posterior regionalization of gene expression in the central nervous systems of three extant bilaterians (an arthropod, an annelid and a vertebrate) and inferred expression in the last common bilaterian ancestor, the urbilaterian. Expression of *Fez* and *Irx* in the annelid *Platynereis* is unknown. For the urbilaterian, both anterior–posterior and medio-lateral gene expression domains are shown. Hypothetical posterior limits of *Irx* and *Gbx* domains in the urbilaterian brain are highlighted by a “?” and dashed lines. PC, protocerebrum; DC, deutocerebrum; TC, tritocerebrum; VC, ventral nerve cord; CG, cerebral ganglion; SG, segmental ganglia; FB, forebrain; MB, midbrain; HB, hindbrain; SC, spinal cord. Gene expression domains based on [1,2,9,24,29,34,42,64],[68-80].

Compatible with a single origin of the CNS, expression of the genes mediating D/V patterning within the CNS is also conserved between protostomes and deuterostomes [66] (Figure 3). These genes are not comparably expressed in cnidarians, suggesting that they were recruited for roles in D/V patterning the CNS of an ancestral bilaterian. Notably, homologs of some key genes expressed mediolaterally in the neuroectoderm of *D. melanogaster* embryos are expressed in comparable domains in the vertebrate CNS. Thus, the *msh* gene is expressed laterally in the *D. melanogaster* neuroectoderm, with *ind* expressed in an intermediate longitudinal domain and *vnd* expressed in a medial stripe of neuroblasts (reviewed in [7,67]). Vertebrate homologs of these three homeobox genes are comparably expressed in the developing neural tube. Two of the three *msh* orthologs (*Msx1, Msx2, Msx3*) are expressed dorsally (that is, laterally) in the roof plate of the CNS, one of the two *ind* orthologs (*Gsh1*) is expressed in the adjacent zone (alar plate), and one of the two *vnd* orthologs (*Nkx2.2*) is expressed more ventrally (that is, medially) in the basal plate.

Additional evidence for homology of protostome and chordate nerve cords, and thus a bilaterian ancestor with a CNS, comes from neuroanatomy, neuronal function and gene expression. Strausfeld and Hirth found striking parallels between the central complex in the arthropod protocerebrum and the basal ganglia in the ventral forebrain of vertebrates [3]. In particular, the vertebrate striatum and pallidum have similar organization as, respectively, the insect fan-shaped body and ellipsoid body. Both the types of neurons and their connections and the functions of these regions are similar in the two organisms. Taken together, the data from comparative gene expression and anatomy provide relatively strong support for a single origin of the CNS in insects and chordates.

#### Parallels between the brains of annelids and vertebrates

Additional evidence for a single origin of the CNS comes from comparisons between annelids and vertebrates. Not only have parallels been drawn between patterning the *Drosophila* and vertebrate brains, but Arendt and colleagues have also noted similarities between the genetic mechanisms patterning the nervous systems of the annelid *Platynereis dumerilii* and vertebrates [2,81] (reviewed in [4]). The annelid brain varies from species to species, with the brains of some species lacking clear compartments but many others having such features as complex, neuron-rich mushroom bodies (a comparatively large part of the brain in insects and annelids that integrates olfactory information) [82]. Extensive comparisons of gene expression have been used to argue for homology between the mushroom bodies and the pallium of the vertebrate brain [2,81]. For example, *Bf-1* (*FoxG1*) is expressed in the anterior part of the vertebrate telencephalon and the pallium as well as in the tip of the annelid brain, while *Wnt5/8* is expressed in the vertebrate pallium and in the annelid mushroom bodies, flanking more medial expression of *Hh* in both [2].

Furthermore, in *P. dumerilii Six3* and *Otx* are expressed anteriorly in the CNS (the peristomium) with the *Six3* domain extending anterior to that of *Otx*[42]. The posterior limit of the *Otx* domain abuts that of *Gbx* in the first larval segment, while the anterior boundaries of *Hox1* and *Hox4* are in the second and third larval segments [48]. *Six3/6* and *Otx* are similarly expressed in acoel flatworms [26,83,84], and in *D. melanogaster* all three genes are expressed in similar patterns as in *P. dumerilii*. Therefore, the annelid cerebral ganglion has been homologized with the insect protocerebrum. In addition, similar to the CNS in *Drosophila* and vertebrates, the neuroectoderm in *P. dumerilii* is divided into a series of domains with outer/dorsal expression of *Msx* and *Pax3/7* (*gooseberry*), intermediate expression of *Nk6* and *Pax6*, and medial expression of *Nkx2.1*/*Nkx2.2*[1].

Together with anatomical similarities, these data showing distinct similarities in expression of genes patterning the CNS both anteriorly/posteriorly and medio/laterally between both major lineages of protostomes (Ecdysozoa and Lophotrochozoa) and vertebrates support a single origin of the CNS in the bilaterian ancestor (Figure 2). The counterargument would be that the CNS in protostomes evolved independently coopting A/P and D/V patterning mechanisms from an ancestor that used them to pattern a body axis. However, this would mean that the extensive similarities in neuronal architecture between chordates, arthropods and annelids would have been convergently evolved, which seems most unlikely.

### Was there a dorso/ventral inversion, and if so, when did it occur?

If the CNS evolved just once, then a D/V inversion must have occurred during evolution of either protostomes or deuterostomes (reviewed in [27,85]). At present, the chief theories are as follows. The first is Anton Dohrn’s idea that a D/V inversion occurred either at the base of the protostomes or within the deuterostomes [86]. The second is the idea most recently articulated by John Gerhart, Christopher Lowe and colleagues that the ancestral deuterostome was hemichordate-like with dorsal and ventral nerve cords and an ectodermal nerve net, with the chordate CNS arising directly from the nerve net [9,24,27] or alternatively, as proposed by van Wijhe [87] and more recently by Nomaksteinsky and colleagues [88], from the dorsal nerve cord. A third theory that the ancestral bilaterian had multiple nerve cords, with one evolving into the protostome CNS and another into the deuterostome CNS, was suggested by Gerhart [27] but has received little attention.

Major evidence supporting a D/V inversion in either basal deuterostomes, basal protostomes or basal chordates is that genes involved specifying polarity of the D/V body axis are expressed in opposite orientations in protostomes and chordates. Sasai, de Robertis and colleagues found that in both groups, BMP signaling is involved in establishing D/V polarity and in neural specification, with suppression of BMP signaling being a prerequisite for formation of a CNS [22,23,89]. In agreement with a D/V inversion having occurred in either the deuterostome or protostome lineages [23], *BMPs* are expressed dorsally in protostomes and hemichordates and the *BMP* antagonist *short gastrulation* (= *chordin* in deuterostomes) is expressed ventrally, while in chordates it is the opposite – *BMPs* are expressed ventrally and *chordin* dorsally. In most bilaterians, D/V orientation of the body and position of the nerve cord are coupled; however, Hejnol and Martindale have noted that expression of *BMP2/4* dorsally (opposite the future mouth) in an acoel with neurite tract(s) dorsally as well as laterally [26] supports the idea that a role for *BMP*/*chordin* in axial patterning may have preceded a role in neural patterning. Another line of evidence supporting D/V inversion comes from analysis of genes involved in left–right patterning. For example, two key regulators of this distinction, *Nodal* and *Pitx*, are expressed on the left side of chordates, but on the right in echinoderms and in some molluscs [90,91].

In summary, conserved expression of some genes along the longitudinal axis of cnidarians and in the CNS and general ectoderm of bilaterians indicates likely cooption of roles for these genes in patterning the CNS. However, similar expression of genes involved in both D/V patterning of the CNS and in A/P regionalization of the brain in chordates and protostomes together with neuroanatomical parallels provides considerable support for the idea that the bilaterian ancestor had a CNS, which was modified or possibly lost in various protostome and deuterostome lineages.

### Hemichordate theories

Despite considerable evidence in support of a single origin of the CNS, data from hemichordates have been interpreted as indicating that the ancestral deuterostome had a nerve net, and therefore the CNSs in chordates and protostomes evolved independently. Hemichordates and echinoderms form a clade, the Ambulacraria, which branched off the deuterostome tree as a sister group to chordates. Indirect developing members of both groups have similar pelagic larvae with an apical tuft of cilia. Echinoderms, which have pentamerous symmetry, typically have an ectodermal nerve net plus radial nerves and a circumoral nerve ring. While Haag proposed that the sea urchin radial nerves are homologous to the chordate CNS [33], most authors disagree [92,93]. Importantly, echinoderm nerve cords do not express *Hox* genes [51,94,95], and an extensive screen by Sly and colleagues for expression of neural patterning genes in a juvenile sea urchin failed to find evidence that the nerve ring or radial nerves are homologous to any part of the brain or nerve cord in bilaterians [93]. Moreover, *Engrailed* is very broadly expressed in the nervous systems and other tissues of the juvenile starfish and not in localized domains as in the chordate CNS [94]. Therefore, echinoderms are currently not considered relevant to the question of evolution of chordate nerve chords. In contrast, the worm-like enteropneust hemichordates, which have longitudinal nerve cords as well as a nerve net, have figured prominently in discussions of the evolution of chordates [10,96-98].

Inferring homologies between chordate nerve cords and hemichordate nervous tissues has been complicated by large differences in morphology. Adult hemichordates have three distinct regions: proboscis, collar and trunk. There are two classes of hemichordates – enteropneusts and pterobranchs. All four families of enteropneusts (Harrimaniidae, Spengelidae, Ptychoderidae, Torquaratoridae) have an ectodermal nerve net, located in all three regions, plus dorsal and ventral nerve cords, suggesting that this organization is a basal hemichordate characteristic. In contrast, the sessile pterobranchs have anterior tentacles and a concentration of neurons at the base of the tentacles that has been termed a brain, as well as several concentrations of neurites and associated neurons extending into the tentacles, the stalk and between the gill slits [99]. Although Romer and others argued that pterobranchs were basal hemichordates [100,101], recent molecular phylogenetic analyses do not distinguish which family is basal [102,103], leaving open the possibility that pterobranchs are derived. Indeed, fossil tube-dwelling enteropneusts from the Cambrian were recently discovered [104].

Most of the work on neural development in hemichordates concerns indirectly developing ptychoderids and the direct developing harrimaniid *Saccoglossus kowalevskii* (reviewed by Röttinger and Lowe [105])*.* Miyamoto and colleagues showed that the larval nervous system in indirect ptychoderids does not carry over into the adult; in late larvae, the larval nervous system is gradually replaced by the adult one [106]. Therefore, it is the development of the adult nervous system that is pertinent for understanding evolution of the CNS.

#### Hemichordates and the argument of an ectodermal nerve net versus a CNS: theory one

There are two competing theories concerning the evolutionary relationship between the nerve net and nerve cords of hemichordates and the chordate CNS (Figure 1). One theory, most recently articulated by Kaul and Stach [107], proposes that one of the hemichordate nerve cords, typically the dorsal one, is homologous to the chordate CNS. This theory implies that the ancestral deuterostome and perhaps also the ancestral bilaterian had a CNS. The chief basis for this idea is that the collar nerve cord neurulates, suggestive of neurulation in vertebrates [88,108]. Anterior and posterior to the collar, the nerve cord is continued by basiepithelial tracts of neurites, which are concentrated dorsally [99]. However, there is nothing that resembles a brain. In the direct developing S. *kowalevskii*, neurulation in the collar nerve cord progresses from posterior to anterior, and there are posterior and anterior neuropores [107]. The nerve cord continues posteriorly as a superficial tract of nerve cell bodies overlying nerve cell fibers and rostral of the anterior neuropore as a wide, superficial tract of both neurons and nerve fibers [88]. In addition to the longitudinal nerve cords, there is a peribranchial nerve ring, which develops from ventral to dorsal, as well as a collar nerve ring at the collar–trunk boundary. Although initially neither nerve cord was thought to contain nerve cell bodies, studies with electron microscopy and with specific nerve cell markers have demonstrated nerve cell bodies and glia in the dorsal nerve cord and at least some neurons associated with the ventral one [107,109,110]. Ventrally in the dorsal cord, there is a neuropil. Bullock [111] and Brown and colleagues [110] have suggested that the large neurons may be homologous to Mauthner cells of the lamprey and Rhode cells of amphioxus. The developing collar and ventral nerve cords as well as the peripharyngeal cord of both *Ptychodera flava* and *S. kowalevskii* express nerve cell-specific genes including *Elav*, *synaptogamin* and also genes for peptides and proteins specific for subsets of nerve cells including *VAChT*, *serotonin*, *Hb9*, *Drg11* and *GABA*. Serotonergic neurons are restricted to the peripheral nervous system, while those labeling with *Drg11*, *Hb9* and cholinergic neurons are preferentially in the collar nerve cord [88,112].

Although most of the ptychoderid ectoderm is non-neural [88], basiepithelial nerve cells are moderately numerous in the proboscis [106]. Nomaksteinsky and colleagues suggested that the more even distribution of neurons in the basiepithelial nerve net of *S. kowalevskii* might represent a transient larval nervous system and that part of the diffuse nervous system of developing *S. kowalevskii* larvae, especially in the proboscis, will become the peripheral nervous system [88]. Based on the morphological and gene expression data, they concluded that it was ‘implausible that the enteropneust skin is homologous to the chordate CNS’. Instead, they argued that the relatively few neurons in the adult “non-neural” ectoderm constituted a peripheral nervous system, and that either the dorsal or ventral nerve cord (they could not decide which one) was homologous to that of chordates [88]. A comprehensive study of developmental gene expression in the developing nerve cords of hemichordates is sorely needed; to date only a few pictures showing expression of genes including *Dlx*, several *Hox* genes, *Tbx2/3*, *PoxN*, *Pitx* and *Olig* in the dorsal and/or ventral midline of embryos of *S. kowalevskii* have been published [9,24,25,113], but whether the tissue expressing these genes is the developing nerve cord or overlying ectoderm is not clear. There are no studies of developmental gene expression in indirectly developing species such as *P. flava* due to a long pelagic larval period [114,115].

A problem with homologizing the dorsal nerve cord of hemichordates with the chordate CNS is the finding by Lowe and colleagues [113] that, as in protostomes, *BMP2-4* and *BMP5-8* are expressed dorsally in *S. kowalevskii*, while *chordin* is expressed ventrally. Consistent with a role in D/V patterning, excess BMP4 protein radializes the embryos and eliminates *chordin* expression, indicating an evolutionarily conserved role of BMP in D/V patterning. This suggests that if either nerve cord in hemichordates is homologous to the chordate CNS, it is the ventral nerve cord, which does not neurulate, and a D/V inversion occurred at the base of the chordates. This is consistent with the gill slits being dorsal and the stomochord, a dorsal/anterior extension of the gut, having been shown to be unrelated to the notochord [116]. Confusing the issue further, in amphioxus and vertebrates, *Nodal* expression dorsally acts in opposition to *BMP* expression ventrally [117], while in sea urchin embryos, BMPs and Nodal oppose each other in patterning the oral/aboral axis (Nodal ventralizes; BMP dorsalizes), suggesting that a role for Nodal in opposing BMPs was present at the base of the deuterostomes and that a D/V inversion occurred in chordates. To some extent this is similar in *S. kowalevskii*, in that perturbation of Nodal signaling results in D/V patterning defects [118]. However, treatment with the Nodal inhibitor SB431542 eliminates expression of both *BMP2/4* and *chordin* and anteriorizes embryos, indicating that Nodal posteriorizes embryos, the opposite of the situation in chordates [118]. These results suggest that the role of Nodal [119] may have been altered in *S. kowalevskii*. Whether a role for BMP/Nodal opposition in D/V patterning was present in the ancestral bilaterian is uncertain. Nodal is involved in left/right patterning in a mollusk [120], but possible roles in D/V or A/P patterning in protostomes have apparently not been investigated. In summary, since it neurulates, the dorsal nerve cord of hemichordates has been proposed as homologous to the chordate CNS. However, the rather scanty data on gene expression are more compatible with homology of the ventral nerve cord and the chordate CNS. More data are clearly needed on both anatomy and gene expression in the hemichordate nerve cords.

#### Hemichordates and the argument of an ectodermal nerve net versus a CNS: theory two

In spite of the evidence supporting the idea that the ancestral bilaterian had a CNS, there is an alternative theory – namely that the ancestral bilaterian and the ancestral deuterostome had ectodermal nerve nets, from which the chordate CNS evolved. The dorsal and ventral nerve cords of hemichordates are therefore not only a hemichordate invention, but are unrelated to the chordate CNS (reviewed in [34]). This theory, most recently articulated by Lowe and colleagues [9,24,113], is based on developmental gene expression and gene interactions in the direct-developing hemichordate, *S. kowalevskii*. It proposes that the chordate CNS evolved from the ectodermal nerve net of a hemichordate-like ancestral deuterostome and maintains that the hemichordate nerve net contains signaling centers evolutionarily related to the anterior neural ridge (ANR), the ZLI and the isthmic organizer (ISO) in vertebrates. As a corollary, part or all of these signaling centers have been lost in the invertebrate chordates (amphioxus and tunicates) [9]. This idea deserves careful consideration because it not only argues that the considerable similarities of gene expression in protostome and chordate nerve cords represent convergent evolution, but it assigns a key position to hemichordates in evolution of the vertebrate CNS.

de Beer [121] was one of the first to recognize the hierarchical nature of homology when he noted similar morphological features in two different animals could develop under the control of different genes; a phenomenon now known as genetic piracy [122]. The converse is also known – where parts of homologous gene networks are involved in the development of apparently nonhomologous structures [123]. When such disconnects are discovered, some would pay more attention to structure [124], and others would pay more attention to the genes (as a deep homology) [125]. In discussing neural evolution in higher deuterostomes, Lowe and colleagues strongly favor genes over morphological features as arbiters of homology. Thus, they maintain that structures with very different morphology (for example, the proboscis of a hemichordate and the forebrain of a vertebrate) are not morphologically homologous [25,126] even though conserved gene expression patterns may indicate a common evolutionary origin. Thus, while those authors find that homologous genetic programs operate at the anterior end of the hemichordate proboscis and vertebrate ANR, at the boundary between the hemichordate proboscis and collar and at the vertebrate ZLI, and at the boundary between the hemichordate collar and trunk and at the vertebrate ISO located at the MHB, they do not refer to these three regions of the hemichordate ectoderm and vertebrate brain as homologs [9]. Even so, as discussed by Wagner [16], these regions could be homologous even though morphologically divergent.

Lowe and colleagues, in their series of papers concerning genes and structures involved in neural evolution of deuterostomes, have been somewhat inconsistent in their treatment of the subject of homology. In their initial work, which concerned expression of 22 genes with restricted ectodermal domains along the A/P axis (Table 1), they concluded that the surface ectoderm of *S. kowalevskii* and the chordate CNS have a common ancestry [24]. These authors noted that *S. kowalevskii* ‘shows pervasive neurogenesis with no large, contiguous non-neurogenic subregion, as occurs in chordates’ and concluded that the deuterostome ancestor had a diffuse ectodermal nerve net that evolved into the vertebrate CNS [24]. One difficulty with this argument is that, as Aronowicz and Lowe [25] later noted, the surface ectoderm of invertebrate chordates outside the neural tube contains widespread ectodermal sensory neurons (Figure 1) [127,128], while in vertebrates the large pan-placodal region outside the neural plate is highly neurogenic [68]. In addition, although the authors maintained that most of 22 genes they studied are not expressed in the “epidermal ectoderm” in chordates [24], in the 10 years since this paper was published, it has become clear that all except possibly *Vax*, *Rx* and *Nkx2-1* are expressed in ectodermal sensory cells in chordates (Table 1) [68,129-131]; expression of *Vax* in amphioxus and of *Nkx2-1* in the tunicate *Ciona intestinalis* have not been determined. For example, *Sox1/2/3* (*SoxB*) and *Hu/Elav* are expressed in ectodermal sensory cells in chordates [129,130], *Hox1*, *Hox3* and *Hox4* are expressed in nested patterns in the amphioxus ectoderm and at especially high levels in ectodermal sensory cells [132], ascidian *Hox1* is expressed in development of ectodermal sensory cells [133], and some *Hox* genes are expressed in placode derivatives in vertebrates [134]. In addition, *Gbx* is expressed in the “non-neural” ectoderm in amphioxus [135] and in developing placodes in vertebrates [136], while *Gbx* and *Otx2* mutually repress one another in development of the otic placode as they do in A/P patterning of the CNS [137]. Therefore, as noted above, the ectodermal expression patterns of genes along the A/P axis do not clearly distinguish CNS from ectoderm outside the CNS. Indeed, Aronowicz and Lowe noted that genes such as *Otx* and *Hox* appear to be involved in patterning neurogenic tissues generally [25]. Thus, expression of these A/P patterning genes alone does not distinguish between theories that the ancestral bilaterian and ancestral deuterostome had an ectodermal nerve net or whether they both had a CNS. As noted above, genes expressed exclusively in the CNS of bilaterians (for example, *earmuff/Fezf/Fezl* and D/V patterning genes) are generally more informative for inferring the course of evolution of the CNS [64]. Indeed, domains of homologs of three genes mediating lateral to medial (D/V) patterning in the chordate and protostome CNSs (*Hh*, *Nkx2.2*, *Msx*) [138-140] are not expressed in comparable patterns in the hemichordate ectoderm [113]. Thus, if the chordate CNS evolved from a nerve net in the ancestral deuterostome, expression of these lateral to medial genes would represent convergent evolution in the CNSs of protostomes and chordates.

**Table 1:** **Gene expression in nervous tissues of -*Saccoglossus kowalevskii *, *Branchiostoma floridae*, *Ciona intestinalis *and vertebrates**

**Gene**	***S. kowalevskii***		***B. floridae***		***C. intestinalis***		**Vertebrate**		**References**
	**s.e.**	**CNC**^**a**^	**s.e.**	**CNS**	**Ectoderm**	**CNS**	**Placode**	**CNS**	
*SoxB*	+	+	+	+	+	+	+	+	[131]
*Hu/Elav*	+	+	+	+	?	?	+	+	
*Nrp/Musashi*	+	+	+	+	?	+	+	+	[141]
*Vax*	+	–	?	?	gene	lost	–	retina, forebrain	[142]
*Rx*	+	–	–	+	–	+	–	+	[69,76]
*Six3*	+	+/−	+	+	–	+	–	+	[43,131,143]
*Nkx2.1*	+	–	–	+	?	?	–	+	[140,144]
*Bf-1*	+	+/−	+	+	+	?	+	+	[131,145,146]
*Dlx*	+	+	+	+	+	+	+	+	[77]
*Pax6*	+	+	+	+	–	+	+	+	[75]
*Tll/Tlx*	+	+/−	+	+	?	?	+	+	[147,148]
*BarH*	+	+	?	?	?	?	+	+	[149]
*Emx*	+	+	?	?	?	?	+	+	[150]
*Otp*	+	?	?	?	+	+	?	+	[131,151]
*Dbx*	+	+	?	?	?	?	–	+	[152]
*Lim1/5*	+	+	+	+	?	?	+	+	[153]
*Irx*	+	+	+	+	?	?	+	+	[154]
*Otx*	+	+	–	+	+	+	+	+	[78,137]
*En*	+	+	+	+	–	+	+ lamprey	+	[78,155]
*Gbx*	+	+	+	+	gene	lost	+	+	[142]
*Hox1*	+	+	+	+	+	+	+	+	[156-158]
*Hox3*	+	–	+	+	+	+	+	+	[134,156,158]
*Hox4*	+	?	+	+	–	–	–	+	[156]
*Hox7/8*	+	?	?	?	gene	Lost	–	+	[156]
*Hox11/13*	+	–	?	?	Hox12+	Hox12+	Hox11+	+	[156,159]

Gene expression in ectoderm and nervous tissue of the hemichordate *Saccoglossus kowalevskii*, the cephalochordate *Branchiostoma floridae*, the tunicate *Ciona intestinalis* and vertebrates. Note: En expression in placodes only documented for lamprey among the vertebrates. CNC, collar nerve cord; CNS, central nervous system; s.e., surface ectoderm. ^a^In the absence of sections, which would distinguish between expression in the surface ectoderm and in the collar nerve cord, all genes with ectodermal expression in the region of the dorsal portion of the collar are listed.

The similarities in patterning the ANR and the anterior part of the hemichordate proboscis, the ZLI and the boundary between the proboscis and collar, and the ISO and the boundary between the collar and trunk lead to the conclusion that in the ancestral deuterostome the role for these signaling centers, which ultimately gave rise to the vertebrate ANR, ZLI and ISO, was to regionalize the general body plan [9]. However, Pani and colleagues also proposed that amphioxus partially lost the ANR, and completely lost both the ZLI and ISO, while the tunicate *C. intestinalis* lost the ZLI and partially lost both the ANR and ISO [9]. They conclude that in ‘certain cases hemichordates will be a more informative group than basal chordates for reconstructing stem chordate characters and understanding the origins of vertebrate developmental genetic processes’ [9]. As this is quite an extreme view and is at odds with conclusions based on morphology, gene expression and gene function in amphioxus, tunicates and vertebrates, the evidence merits close examination.

#### How much of the ANR gene network is present in S. kowalevskii? Is the evidence sufficient that the vertebrate ANR evolved from the anteriormost ectoderm of the ancestral deuterostome?

**Figure 4:**
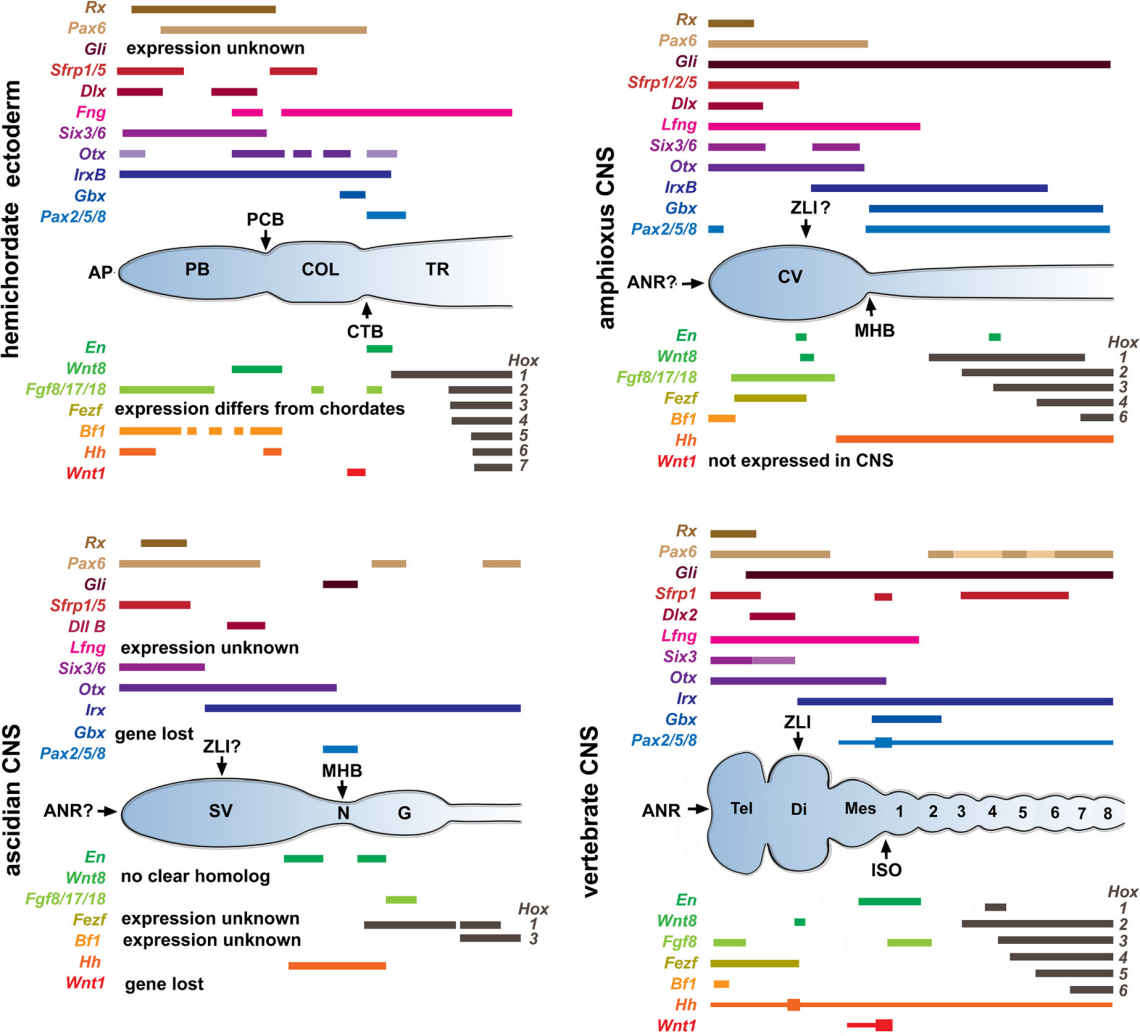
**Anterior–posterior gene expression in hemichordate ectoderm and central nervous systems of three chordate subphyla.** Anterior–posterior regionalization of gene expression domains in the ectoderm of the hemichordate *Saccoglossus kowalevskii* as well as in the central nervous system (CNS) of representatives of the three chordate subphyla (that is, amphioxus, ascidian tunicates and vertebrates). Question marks on the diagrams of the amphioxus and ascidian CNS indicate that organizer properties of the regions marked ‘ZLI?’ and ‘ANR?’ have not been tested. AP, anterior proboscis; PB, proboscis; PCB, proboscis/collar boundary; COL, collar; CTB, collar/trunk boundary; TR, trunk; ANR, anterior neural ridge; ZLI, zona limitans intrathalamica; MHB, midbrain/hindbrain boundary; SV, sensory vesicle; N, neck; G, ganglion; ISO, isthmic organizer; Tel, telencephalon; Di, diencephalon; Mes, mesencephalon. Gene expression domains based on [9,24,34,64,68-72,74-80].

The vertebrate ANR is characterized by expression of *Fgf8*, *Six3*, *Pax6*, *Otx2*, *Sox2* with *Dlx5* expressed in adjacent non-neural ectoderm and *Bf-1* (*FoxG-1*) expressed in the rostral forebrain (Figure 4) [160], while *Sfrp1a* is expressed in the anterior/ventral part of the developing neural tube [161]. In addition, transplantation of the ANR laterally expanded the telencephalon and promoted expression of *Bf-1* (*FoxG1*), demonstrating that the ANR is an organizer [162]. In *S. kowalevskii*, homologs of these genes are expressed in the proboscis ectoderm, although their relative domains are rather divergent from those of their homologs in the vertebrate forebrain. For example, *Six3*, *Sfrp1/5* and *Fgf8/17/18* are strongly expressed in the anterior proboscis ectoderm, and *Sox1/2/3* is broadly expressed in the proboscis and anterior part of the collar [9]. However, *Pax6* is not expressed at all in the anteriormost proboscis ectoderm but more posteriorly throughout much of the proboscis and collar, while *Otx* is chiefly expressed in the collar ectoderm with only very weak expression at the anterior end of the proboscis. In addition, *Dlx* is very patchily expressed in the proboscis ectoderm, but appears to be highly expressed where the dorsal nerve cord will form. *Hh* is expressed in ectoderm at the tip of the proboscis in *S. kowalevskii*[9], while in vertebrates it is expressed in the basal plate of the forebrain and midbrain and in the floor plate of the anterior hindbrain as well as in the prechordal plate [163].

Experimental evidence for homology of gene networks patterning the ANR and anterior proboscis ectoderm in *S. kowalevskii* came from manipulation of Fgf and Hh signaling. Although inhibition of *Fgf* or Fgf signaling scarcely affected expression of the proboscis domain of the anterior marker *Rx*, it did eliminate *FoxG1* (*BF-1*) expression in the proboscis. In addition, knockdown of *Hh* eliminated expression of the anterior marker *Fgf-Sk1*. Thus, inhibition of Fgf or Hh signaling affects development of the proboscis and expression of some anterior markers. However, Green and colleagues found that the major role of *Fgf8/17/18* in development appears to be in mesoderm induction [164]. Therefore, the effects of inhibition of Fgf signaling on anterior development could be secondary to those on mesoderm.

Whether this evidence is sufficient to indicate that the vertebrate ANR evolved from the anterior part of a diffuse ectodermal nerve net in the deuterostome ancestor is open to question. A major difficulty in identifying homologous gene networks is deciding how much of two gene networks must be conserved for them to be considered homologous [165]. This is a particular problem when the morphology is not conserved. Gene networks can include several thousand genes, and it is well known that core parts of signaling pathways are often coopted for patterning nonhomologous structures [166]. Thus, there are several alternative explanations for similarities in gene expression between the hemichordate proboscis ectoderm and the vertebrate ANR. One is that similar expression of a suite of genes including *Fgfs* in these two regions may simply be indicative of ancient roles in specification of the anterior end of embryos in general. For example, Sinigaglia and colleagues reported that *Six3/6*, *FoxQ2*, *Irx*, *SoxB1* and *Fgf* are expressed in the aboral region in the cnidarian *N. vectensis*, while *Wnts* are expressed at the opposite end of the embryo [39]. *Six3/6* is initially involved in specification of the anterior end of the embryo and later in neurogenesis. Fgf signaling is required for development of the apical tuft of cilia. Therefore, roles for these genes in patterning the hemichordate proboscis may simply reflect an inheritance from prebilaterians. Another possibility is that if, as comparisons of protostomes and chordates indicate, these genes were coopted for patterning the anterior CNS of the ancestral bilaterian, then the ancestral deuterostome might have had a more extensive CNS than modern hemichordates. If so, the forebrain may have been lost as the proboscis evolved, and the genetic pathways for anterior neural development coopted into the proboscis ectoderm. Expression of *Rx*, *Fgf8/17/18* and other genes in the hemichordate proboscis is compatible with either theory. In chordates, *Rx* expression is apparently restricted to the anterior end of the forebrain (it is not expressed in sensory ectoderm) [69], while *Fgf8/17/18* is expressed in the ANR and telencephalon of vertebrates as well as throughout the forebrain of amphioxus. In contrast, *Hh* is expressed in the anterior tip of the notochord in amphioxus and in the floorplate but not in the anterior CNS. Given that *Fgf8* and *Hh* have been coopted in vertebrate limbs, which evolved in gnathostomes, for patterning the apical ectodermal ridge (AER) and zone of polarizing activity (ZPA) [167], it is certainly possible that these genes plus *Rx* and other genes involved in neuronal specification may have been coopted for patterning the proboscis ectoderm of hemichordates.

Another possibility, proposed by Nomaksteinsky and colleagues [88], is that the ectoderm of developing *S. kowalevskii* may represent a transient larval nervous system unrelated to that of the adult. Evidence is that *SoxB* genes, which mark the neural plate in chordates, are broadly expressed in the ectoderm of indirectly developing embryos of the hemichordate *Ptychodera flava*, with expression becoming localized during development to the stomodaeum, ciliary bands and apical ciliary tuft [114]. *Dlx* is expressed in the aboral ectoderm, with a concentration towards the apical tuft, and *Fz5/8* and *FoxQ2* are expressed in the apical tuft [168]. Similarly in *S. kowalevskii*, *Six3, Fz5/8* and *Dlx* are broadly expressed in the proboscis ectoderm and *Bf-1* (*FoxG1*) in the apical region [9,24]. The tornaria larva of indirect developing hemichordates has much in common with the dipleurula type of echinoderm larvae, which also have an apical tuft and a band of cilia around the mouth. Moreover, gene expression in the apical tuft and surrounding ectoderm is highly conserved in the sea urchin and hemichordate larvae, and there is considerable similarity with expression in the proboscis ectoderm of *S. kowalevskii* embryos [169]. In sea urchins, *FoxQ2*, *Fzl5/8*, *Sfrp1/5*, *Dkk3* and *Six3* are expressed at the apical end of the larva, with Wnt signaling acting to restrict expression of these genes to the anterior end of the larva [169,170]. Similarly, in *S. kowalevskii* knocking down the Wnt receptor *Fz5/8* results in a posterior expansion of the domains of anterior markers [9], indicating that Wnt signaling is also regulating expression of genes in the proboscis ectoderm as it is in the apical ectoderm of sea urchin larvae.

#### How much of the ZLI gene network is present in S. kowalevskii Does the evidence support evolution of the vertebrate ZLI from a boundary partitioning the anterior/posterior axis of the ancestral deuterostome?

The vertebrate ZLI is positioned and regulated, at least in part, by anterior expression of *Otx* and *Fezf* abutting a posterior domain of *Irx*[171,172]. Organizer properties are conferred by *Hh*, which is expressed at the ZLI and which is regulated by *Wnt8b* (Figure 4) [173]. In fish, knockdown of *Fezf2*, which is expressed anterior to *Irx*, eliminates the prethalamus and causes mis-specification of the ZLI. Correspondingly, knockdown of *Otx* inhibits expression of *Shh* at the ZLI and reduces expression of *Ptc1* and *Wnt8b*[172]. Evidence for organizer properties of the vertebrate ZLI comes from grafting experiments and implants of Fgf8-coated beads, which induce the rostral neuroepithelium to develop an ectopic and polarized mesencephalon/metencephalon (reviewed in [174]).

The region of the hemichordate ectoderm expressing homologs of ZLI genes lies at the boundary between the proboscis and the collar [9] and is marked by the posterior limit of *Rx* expression and bands of *Fgf8/17/18*, *FoxG*, *Otx*, *Wnt8*, *Hh*, *Ptch*, *FoxA* and *Dlx*. Congruent expression of *Wnt8* and *Hh* in *S. kowalevskii* is consistent with a possible role of *Wnt8* regulating *Hh* as it does at the ZLI in vertebrates. However, domains of *Fezf* and *Irx*, which abut at the ZLI in vertebrates, do not abut at the proboscis/collar boundary in *S. kowalevskii.* Expression of *Fezf* was not shown, but the major domain of *Irx* appears to be congruent with that of *engrailed* in the posterior part of the collar [24].

For *S. kowalevskii*, the experimental evidence presented by Pani and colleagues for the proboscis/collar boundary region acting as a signaling center was that knockdown of *Otx* expression reduced the intensity of the stripe of *Hh* expression just posterior to the proboscis/collar boundary, while inhibition of Hh signaling downregulated *Dlx* both at the proboscis/collar boundary and more anteriorly and also reduced the size of the proboscis [9]. These results show that *Otx* may act upstream of *Hh* at the proboscis/collar boundary, while *Hh* is vital for normal development. However, *Otx* and *Shh* also interact in patterning the vertebrate midbrain, not just the ZLI, and knockdown of *Otx* results in dorsal expansion of the *Shh* domain and a dorsal and anterior rotation of the MHB [175]. Thus, the relationship between *Otx* and *Hh* and perhaps some other parts of the gene network may be evolutionarily conserved between *S. kowalevskii* and vertebrates, but whether tissue at the proboscis/collar boundary in an ancestral deuterostome evolved into the ZLI is open to question given the markedly different expression of key genes such as *Fezf* and *Irx* as well as the lack of anatomical similarity.

#### How much of the ISO gene network is present in S. kowalevskii Does the evidence support evolution of the vertebrate ISO from a boundary partitioning the anterior/posterior axis of the ancestral deuterostome?

In the vertebrate CNS, the MHB or isthmus functions as an organizer, and is therefore termed the isthmic organizer or ISO. The MHB is positioned by opposition of anterior *Otx* and posterior *Gbx*. Organizer properties are conferred by the action of a suite of genes including *En*, *Fgf8/17/18*, *Wnt1* and *Pax2/5/8* (Figure 4) [176]. *Otx* and *Fgf8* are expressed in the midbrain and *Gbx* in the anterior hindbrain, with the *Wnt1*, *Engrailed* and *Pax2/5/8* domains spanning the boundary; mutual repression of *Otx* and *Gbx* positions the ISO, and Wnt signaling is required for expression of *Engrailed*. Pani and colleagues also presented evidence for a homologous gene network in *S. kowalevskii* operating at the boundary between the collar and trunk [9]. *Engrailed*, *Fgf8/17/18*, *Wnt1* and *Gbx* are expressed near this boundary. In addition, clonal suppression of *β-catenin* effectively inhibited *Engrailed* expression, suggesting that Wnt signaling regulates *Engrailed*, as it does at the ISO in vertebrates, while suppression of *Fgf8/17/18* reduced *Engrailed* expression at the collar/trunk boundary. However, despite these similarities, there are several problems in interpreting the gene network at the collar trunk boundary in *S. kowalevskii* as homologous to that patterning the vertebrate ISO. Importantly, as the authors note, the ‘spatial arrangements of *Otx* and *Wnt1*, and *Gbx* and *Fgf8/17/18* are reversed in *S. kowalevskii* compared to the ISO in vertebrates’ [9]. Moreover, suppression of Fgf signaling had no effect on expression of *Pax2/5/8.* A problem in interpreting these experiments is that in vertebrates, *Fgf8* has an early role in induction of neural tissue, while patterning the ISO is a relatively late role. In *S. kowalevskii, Fgf8/17/18* is expressed from the blastula stage, and by the neurula stage expression is restricted to anterior ectoderm. Knockdown experiments show that *Fgf8/17/18* is required for mesoderm induction [164]. Therefore, effects of *Fgf8/17/18* inhibition in *S. kowalevskii* on *Engrailed* expression may be secondary to the loss of mesoderm and not directly related to effects on A/P patterning. Additional evidence for the common ancestry of the collar/trunk boundary and the vertebrate ISO was that *Hox* genes are expressed in nested patterns in the trunk ectoderm. However, it appears that at least *Hox1* and perhaps *Hox4* are also expressed in the dorsal part of the collar (Figure five in [24]), indicating that Hox expression in the ectoderm is probably not congruent with that in the collar, and raising the question of the genetic mechanisms patterning the collar nerve cord and how they might compare with those in chordates.

As for the ANR and ZLI, drawbacks to interpreting the boundary of the collar and trunk in hemichordates with the vertebrate ISO as having common ancestry not only include differences in expression of key genes expressed in these regions and also the general phenomenon of cooption of parts of gene networks for new functions. For example, in the AER of the vertebrate limb bud, *Wnt3a* induces expression of *Fgfs*, while ectopic *Engrailed* (*En-1*) induces ectopic *Fgf8* expression [177,178]. Moreover, in amphioxus, *engrailed* is co-expressed with *Wnt8*, but not at the MHB, suggesting that coexpression of *Wnt* and *engrailed* is not wedded to the MHB. The questions therefore become: how conserved must gene networks be in order for one to be reasonably certain that two morphologically rather different structures have a common ancestry; and if they do share an ancestry, can one distinguish whether portions of the gene networks operating in the CNS of a deuterostome ancestor were transferred to the hemichordate ectoderm as the hemichordate CNS became reduced or whether the ancestral deuterostome lacked a CNS and used these gene networks to partition the A/P axis?

### How much of the ANR, ZLI and ISO do invertebrate chordates have?

In addition to asserting that the *S. kowalevskii* ectoderm is not only homologous to the vertebrate CNS but also has homologs of the ANR, ZLI and ISO, Pani and colleagues maintain that the invertebrate chordates, amphioxus and tunicates, have lost all or part of these three regions [9]. Here we draw attention to data that are not consistent with such a view (Figure 4).

Although transplantation experiments in vertebrates demonstrated that the ANR functions as an organizer [163], such transplantation experiments are not feasible for hemichordate, amphioxus or *C. intestinalis* embryos due to their small size. However, it is clear that much of the gene network for specification of the ANR and conferring organizer properties upon it is present in amphioxus. *Fgf8/17/18* is expressed in the entire forebrain of amphioxus, and *Bf-1* (*FoxG1*) is expressed at the tip of the forebrain [145] as are *Pax2/5/8* and *Six3/6*[43]. *Otx* and *Pax6* are expressed in comparable patterns with strong expression in the anterior forebrain [135,179], and the Wnt antagonist *Sfrp1/2/5* is expressed in the anteriormost dorsal ectoderm, including the most anterior neuroectoderm [180]. Moreover, as in vertebrates, *Dlx* is expressed in ectoderm outside the neural tube as well as in the edges of the anterior neural plate [70], while Hh is expressed in the underlying tip of the notochord [181,182]. Taken together, these expression patterns indicate that amphioxus has most of the components for an ANR comparable to that in vertebrates.

Tunicates have undergone some radical changes to their genome and their anatomy in evolution. For example, the *Gbx* gene has been lost, and there are relatively few neurons – none in the tail nerve cord of ascidians. *Otx* is expressed anteriorly in the CNS, but the evidence for an ANR in *C. intestinalis* is relatively weak. In addition to *Otx*, *Pax6* is expressed in the anterior CNS, but not *Fgf8/17/18* and *Pax2/5/8*, *Hh*, or *Gli*[183]. Expression of *Bf-1* is not known. Therefore, it may be that as tunicates adopted early decision of cell fates and decreased the number of cells in the nerve cord, the need for an anterior brain organizer diminished.

For the ZLI, patterns of gene expression indicate that amphioxus probably has much of the genetic mechanism in place. In amphioxus, Irimia and colleagues showed that anterior expression of *Fezf* abuts posterior expression of *IrxB* about the midpoint of the forebrain [64] – approximately where *Wnt8* is expressed [184]. Similarly, *Wnt8b* expression at the ZLI in vertebrates is flanked by *Lfng*, while in amphioxus the *Fng* domain appears to be posterior to and possibly abutting that of the *Wnt8* domain at the late neurula stage [184,185]. *Dlx*, *Nkx2-2* and *Gli*, which mediates Hh signaling, are also expressed in this region, while *Fgf8/17/18* is expressed throughout the forebrain [70,71,186]. *Hh* is expressed in the floor plate as it is in vertebrates, but it is unclear at the early neurula stage whether it is expressed congruently with *Engrailed* and *Wnt8* or a little more posteriorly. Later expression is limited to a zone posterior to the forebrain [181]. Inhibition of Fgf signaling at the late blastula does not inhibit expression of neural plate markers, but eliminates that of *Otx* in the cerebral vesicle (forebrain) and reduces its size [187], indicating that Fgf signaling is essential for development of the forebrain. Similarly, upregulation of Wnt/β-catenin signaling reduces expression of *Otx* in the forebrain and eliminates expression of the anterior marker *FoxQ2*[188], showing that suppression of Wnt/β-catenin signaling by inhibitors such as Sfrp1/2/5 [180] is essential for forebrain development.

For ascidians, expression of *Irx* and *Fezf* is not known. *C. intestinalis* has two *Wnt* genes that are possibly related to *Wnt8* but which have long branches in phylogenetic analyses and for which assignation remain unclear [189]. Expression of these genes has not been characterized. However, *Gli*, *Hh* and *Fgf8/17/18* are not expressed in the anterior CNS. Even so, inhibition of Fgf signaling blocks expression of the anterior marker *Six3/6*, indicating that Fgf signaling is required for development of the anterior brain [190]. Thus, while *C. intestinalis* likely lacks most of the gene network specifying the vertebrate ZLI and conferring organizer properties, until the expression patterns of *Irx* and *Fezf* are known the presence of part of the network for specification of the ZLI cannot be ruled out.

Amphioxus also appears to have in place the genetic mechanism for positioning the ISO, but this region does not express homologs of the genes that confer organizer properties on the vertebrate ISO. In the amphioxus brain, as in that of vertebrates, the domain of *Otx* expression in the forebrain/midbrain abuts that of *Gbx* in the hindbrain [135]. In vertebrates, organizer properties are conferred on the ISO by expression of *Fgf8*, *Wnt1*, *Engrailed* and *Pax2/5/8* genes at this boundary. In amphioxus, *Wnt1* and *Engrailed* are not expressed between the *Otx* and *Gbx* domains, although *Fgf8/17/18* is expressed anterior to and abutting this boundary, while *Pax2/5/8* is expressed posterior to and abutting it [187,191,192]. *Engrailed* is expressed together with *Wnt8* approximately at the boundary between the *Fezf* and *IrxB* domains. Interestingly, there is also a stripe of *Engrailed* in the general ectoderm that is in register with that in the CNS [193].

The tunicate *C. intestinalis* appears to have more of the ISO network in place than amphioxus does, but it has lost a key gene – *Gbx*. *Pax2/5/8* and *Fgf8/17/18* are expressed in the neck region in a few cells just posterior to the posterior limit of *Otx*. *Engrailed* is expressed in two domains, one near the *Pax2/5/8* domain and the other overlapping with that of *Otx*[194].

The emerging picture is that amphioxus has most, if not all of the genetic mechanism for the ANR in place and part of that for the ZLI and ISO while the ancestral tunicate probably added to these gene networks but subsequently lost parts of them as the CNS became simplified. The most parsimonious explanation is that the ancestral bilaterian had a CNS in which portions of these gene networks were used to establish divisions in the CNS and perhaps to some extent in the ectoderm generally, and these gene networks became modified in the protostome and deuterostome lineages. Under this scenario, the basic gene networks for specifying the ANR, ZLI and MHB began to be established in the ancestral chordate or even earlier in the ancestral deuterostome or ancestral bilaterian, and additional genes were recruited to these networks before tunicates branched off the lineage leading to vertebrates. In the tunicate lineage, in conjunction with shrinking genomes, a switch from regulative to determinate development and a reduction of cell numbers in the CNS, some parts of the ancestral tunicate networks for these brain regions were lost. Modifications occurring in the hemichordate lineage may have involved changes in the CNS and/or cooption of additional genes and portions of gene networks to the surface ectoderm.

## Conclusions

The bilaterian CNS typically develops from a longitudinal strip of ectoderm that is then internalized. Although the remainder of the ectoderm is usually termed “non-neural”, it also contains numerous neurons – often sensory neurons – which send signals to the CNS. Therefore, the ectoderm really should be thought of as “more neural” and “less neural” rather than “neural” and “non-neural”. This is highly relevant for discussion of how a CNS evolved from an ectodermal nerve net, since part of the genetic mechanism(s) for patterning the bilaterian CNS are shared with the ectoderm as a whole.

The basic A/P patterning mechanism in bilaterians with *Six3/6* expressed anteriorly and *Wnt* genes posteriorly was inherited from an ancestral cnidarian-like animal. Additional genes (for example, *Hox* genes) were recruited to pattern the A/P axis of the ancestral bilaterian. Consequently, even though the ectoderm in annelids and arthropods is segmented, and the ectoderm of hemichordates and chordates is not, several genes that mediate A/P patterning of the CNS are expressed in similar patterns along the A/P axis of the non-neural ectoderm in early embryos/larvae of both protostomes, chordates and hemichordates. For example, the patterns of *Otx*, *Gbx* and *Hox* genes in the early ectoderm of the annelid *P. dumerilii* are conserved in the neuroectoderm of amphioxus embryos with an anterior *Otx* domain abutting that of *Gbx*, anterior to nested domains of *Hox* expression [48]. Expression of *Hox* genes and *Gbx* in the “non-neural ectoderm” in amphioxus is similar to that in the CNS [131,134,192]. In contrast, in the hemichordate ectoderm, while *Otx* and *Gbx* domains lie anterior to nested *Hox* domains, that of *Gbx* lies in between two domains of *Otx*[9].

More critical for distinguishing a CNS from the remainder of the ectoderm is the expression of the lateral/medial patterning genes (*msh/Msx*, *ind/Gsh1*, *vnd/Nkx2.2*), because expression of these genes is similar in the CNS of protostomes and chordates, but they are not expressed in comparable patterns in the “non-neural” ectoderm of either group.

The idea that the hemichordate ectoderm has organizing centers patterned by gene networks equivalent to those operating at the ANR, ZLI and ISO in the vertebrate CNS is based on similarities in gene expression as well as in some conserved gene interactions. However, some key genes involved in establishing these organizing centers in the vertebrate CNS are not similarly expressed in the hemichordate ectoderm. For example, in the *S. kowalevskii* ectoderm, the *Fezf* and *Irx* domains, which mark the vertebrate ZLI, do not abut at the collar/proboscis interface, while patterns of *Otx* and *Wnt1* as well as those of *Gbx* and *Fgf8/17/18*, which mark the ISO, are reversed*.* Moreover, since parts of gene networks are often coopted for roles in different tissues, the fact that two genes interact does not suffice to demonstrate that a given region acts as an organizer. Thus, without transplantation experiments or at least the demonstration that a given morphogen such as *Fgf8/17/18* can induce a change in cell fate when ectopically expressed, conclusions that homologs of these organizing centers are present in hemichordates are premature.

In addition, since several genes involved in specification of the ISO and ZLI are expressed in comparable positions in the amphioxus and vertebrate CNS as well as in the protostome CNS, claims that amphioxus has completely lost these regions are unwarranted. A more parsimonious explanation is that amphioxus has part of the machinery in place, upon which tunicates and vertebrates elaborated. Tunicates do appear to have more of the genetic machinery for specification of the ISO than amphioxus does, but have lost some key components, such as the *Gbx* gene.

The Ambulacraria (hemichordates and echinoderms), which are the sister group to chordates in the deuterostomes, are interesting in regard to what evolution can do. Both hemichordates and echinoderms have ectodermal nerve nets plus nerve cords. Gene expression indicates that the nerve cords of echinoderms, which have evolved pentamerous symmetry, are probably not homologous to chordate or protostome nerve cords. *Hox* genes, for example, are expressed in the developing coeloms of the adult, but not in the nerve cords or ectoderm [48,51]. It has been suggested that one or the other nerve cord of hemichordates is evolutionarily related to the chordate CNS, with historical opinions generally favoring the dorsal cord. However, dorsal expression of *BMPs* suggests that this homolog would be the ventral nerve cord even though it is the dorsal nerve cord that neurulates. If this is true, then a D/V inversion would have occurred at the base of the chordate lineage.

In sum, similar expression patterns of developmental genes involved in both A/P and D/V patterning in the protostome and chordate nerve cords as well as anatomical and functional similarities support the view that the ancestral bilaterian had a CNS. In the Ambulacraria, echinoderms may have lost this CNS, while it remains to be seen whether either of the hemichordate nerve cords is homologous to that of protostomes and chordates. Similarities of gene expression in the hemichordate ectoderm and chordate CNS may reflect conservation of A/P patterning in the ectoderm as a whole and in the CNS of bilaterians and/or a merging of the anterior portion of the brain and the proboscis ectoderm during evolution of hemichordates. A comprehensive study of expression of developmental genes in the hemichordate nerve cords would help to resolve these questions. In any event, similarities in gene expression between the CNS of amphioxus and vertebrates indicate that amphioxus has at least part of the genetic mechanisms for the ANR, ZLI and MHB in place and suggest that vertebrates subsequently elaborated upon these gene networks.

## Abbreviations

ANR: Anterior neural ridge; A/P: Anterior/posterior; BMP: Bone morphogenetic protein; CNS: Central nervous system; D/V: Dorso/ventral; ISO: Isthmic organizer; MHB: Midbrain/hindbrain boundary; ZLI: Zona limitans intrathalamica.

## Competing interests

The authors declare that they have no competing interests.

## Authors’ contributions

LZH coordinated and wrote much of the manuscript. MS, HE, SMS, JKY and VL contributed to writing the manuscript. JEC and MS prepared the figures. All authors read and approved the final manuscript.
